# How can digital citizen science approaches improve ethical smartphone use surveillance among youth: Traditional surveys versus ecological momentary assessments

**DOI:** 10.1371/journal.pdig.0000448

**Published:** 2024-11-11

**Authors:** Sarah Al-akshar, Sheriff Tolulope Ibrahim, Tarun Reddy Katapally

**Affiliations:** 1 DEPtH Lab, School of Health Studies, Faculty of Health Sciences, Western University, London, Ontario, Canada; 2 Department of Epidemiology and Biostatistics, Schulich School of Medicine and Dentistry, Western University, London, Ontario, Canada; 3 Children’s Health Research Institute, Lawson Health Research Institute, 750 Base Line Road East, Suite 300, London, Ontario, Canada; University of Pittsburgh School of Medicine, UNITED STATES OF AMERICA

## Abstract

Ubiquitous use of smartphones among youth poses significant challenges related to non-communicable diseases, including poor mental health. Although traditional survey measures can be used to assess smartphone use among youth, they are subject to recall bias. This study aims to compare self-reported smartphone use via retrospective modified traditional recall survey and prospective Ecological Momentary Assessments (EMAs) among youth. This study uses data from the Smart Platform, which engages with youth as citizen scientists. Youth (N = 77) aged 13–21 years in two urban jurisdictions in Canada (Regina and Saskatoon) engaged with our research team using a custom-built application via their own smartphones to report on a range of behaviours and outcomes on eight consecutive days. Youth reported smartphone use utilizing a traditional validated measure, which was modified to capture retrospective smartphone use on both weekdays and weekend days. In addition, daily EMAs were also time-triggered over a period of eight days to capture prospective smartphone use. Demographic, behavioural, and contextual factors were also collected. Data analyses included t-test and linear regression using Python statistical software. There was a significant difference between weekdays, weekends and overall smartphone use reported retrospectively and prospectively (p-value = <0.001), with youth reporting less smartphone use via EMAs. Overall retrospective smartphone use was significantly associated with not having a part-time job (β = 139.64, 95% confidence interval [CI] = 34.759, 244.519, p-value = 0.010) and having more than two friends who are physically active (β = -114.72, 95%[CI] = -208.872, -20.569, p-value = 0.018). However, prospective smartphone use reported via EMAs was not associated with any behavioural and contextual factors. The findings of this study have implications for appropriately understanding and monitoring smartphone use in the digital age among youth. EMAs can potentially minimize recall bias of smartphone use among youth, and other behaviours such as physical activity. More importantly, digital citizen science approaches that engage large populations of youth using their own smartphones can transform how we ethically monitor and mitigate the impact of excessive smartphone use.

## Author summary

Use of ubiquitous digital devices, particularly smartphones, has experienced an exponential increase among youth, a phenomenon that continues to influence youth health. Although retrospective measures have been used to understand smartphone use among youth, they are prone to measurement and compliance biases. There has been a growing interest in using ecological momentary assessments (EMAs) to assess smartphone to minimize biases associated with retrospective measures. This study uses the Smart Framework, which integrates citizen science, community based participatory research and systems science to ethically engage with youth citizen scientists using their own smartphones to understand smartphone use behaviours–reported by the same cohort of youth using both retrospective and prospective measures. The findings show a significant difference between smartphone use reported through retrospective and prospective EMAs, with youth reporting more smartphone use via retrospective measures. Furthermore, there were differences in contextual and behavioural factors that were associated with smartphone use reported via retrospective and prospective measures. The findings have implications for appropriately understanding and monitoring smartphone use in the digital age among youth. More importantly, digital citizen science approaches that engage large populations of youth using their own smartphones can transform how we ethically monitor and mitigate the impact of excessive smartphone use.

## Background

The prevalence of smartphone usage among the younger population has experienced a significant surge in recent years on a global scale, as evidenced by the fact that 95% of smartphone users fall within the 13–17 years [1,2]. Nonetheless, there is still ambiguity regarding how youth use their smartphones and the potential implications of using ubiquitous digital tools such as smartphones [3].

In understanding smartphone use among youth, thus far, traditional self-report measures have been predominantly used [4], which pose issues such as recall bias and measurement errors [5,6]. Moreover, traditional survey measures do not capture the full range of activities associated with smartphone use, including communication, e-learning, entertainment, and social media, among others [6].

Advanced population health measurement techniques such as ecological momentary assessments (EMAs), can potentially address measurement bias and capture the entire range of smartphone activities [7,8]. EMAs “capture brief, repeated assessments in natural environments with a high degree of ecological validity relative to laboratory-based investigations” [9]. EMAs are prospective measures that provide an advantage in assessing behaviours by capturing environmental context of the participants [10]. While traditional surveys are important in understanding behaviour retrospectively, EMAs have the advantage of minimizing recall bias and improving the validity of results [11]. Moreover, EMAs reduce the burden of responding to surveys and improve rates of compliance and feasibility for participants [12], as well as minimize social desirability bias [13].

Although current research examines screen-based behaviours among youth, including watching TV and playing video games, there is a scarcity of evidence focusing on smartphone use [14]. It is critical to appropriately understand smartphone use among youth to not only inform potential smartphone use recommendations [15], but also to determine appropriate use for digital health interventions [16]. In addition, currently no studies exist that compare retrospectively reported smartphone use using traditional survey measures and prospectively reported smartphone use using EMAs within the same cohort of youth. The objective of this study is to compare smartphone use reported via retrospective surveys and prospective EMAs by engaging with youth citizen scientists via their own smartphones. The study also investigates the demographic, behavioural, and contextual factors that are associated with smartphone use reported via retrospective surveys compared to prospective EMAs.

## Methodology

### Study design

This study is a part of the Smart Platform, which is a citizen science and digital epidemiological initiative for ethical population health surveillance, knowledge translation, and real-time interventions [17]. Smart Platform integrates community-based participatory research and digital citizen science to ethically obtain big data from citizen-owned smartphones [17]. Moreover, the study uses a combination of cross sectional and temporal longitudinal design [18].

**Fig 1:**
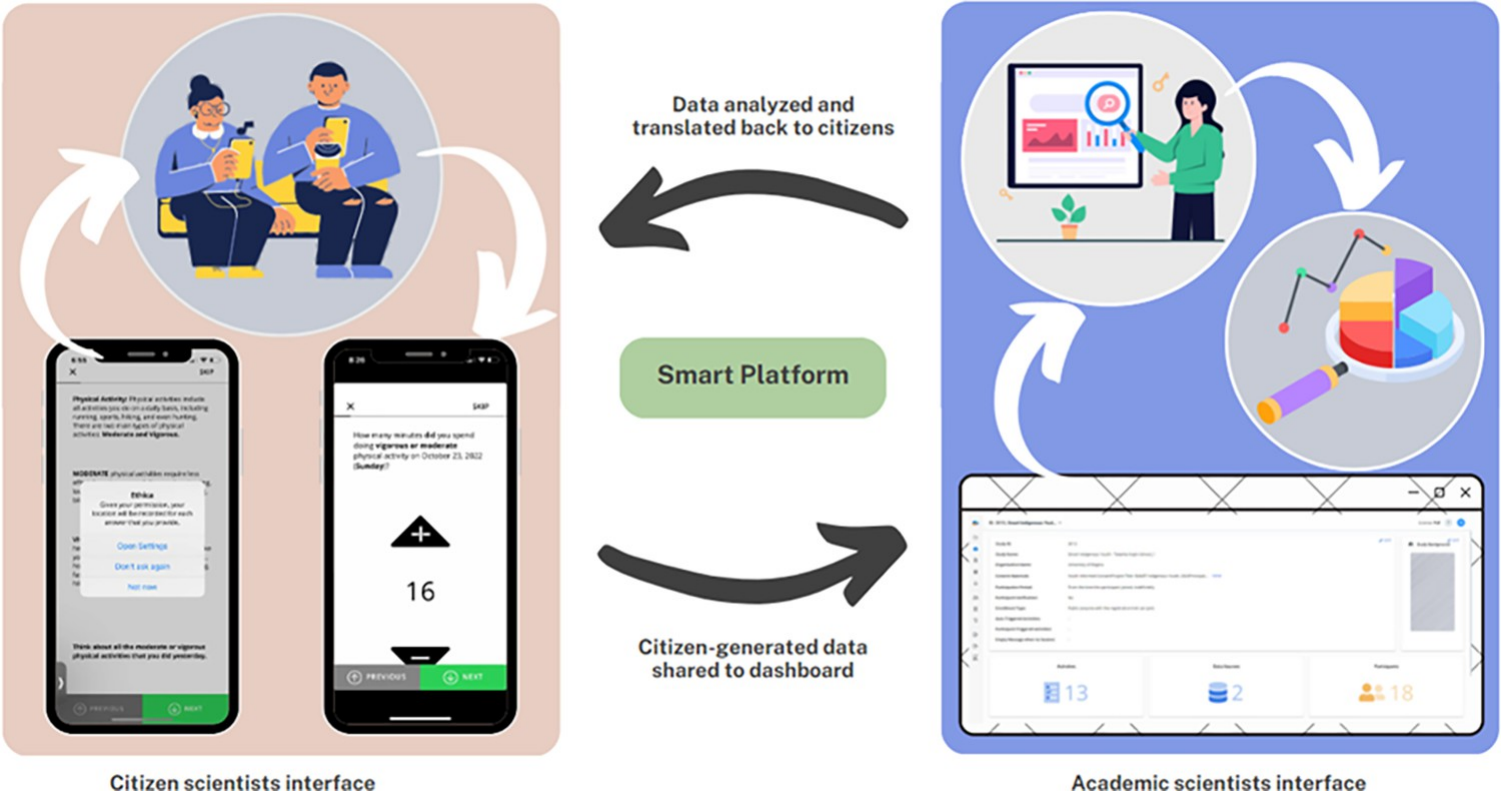
The Smart Platform: citizen scientists’ interface + academic scientists interface.

The research team engaged with youth citizen scientists via their smartphones over the course of eight days through the Smart Platform smartphone custom-built application. Youth citizen scientists could download the application through the iOS or Android operating systems [18] **Fig 1** illustrates the interfaces utilized by both citizen scientists and academic scientists within the Smart Platform. It highlights the flow of data from the citizen scientists during data collection to the analysis done by the academic scientists, the findings from which are in turn translated back to the citizen scientists through the smartphone app, emphasizing the central role of the Smart Platform in this engagement.

**Fig 2:**
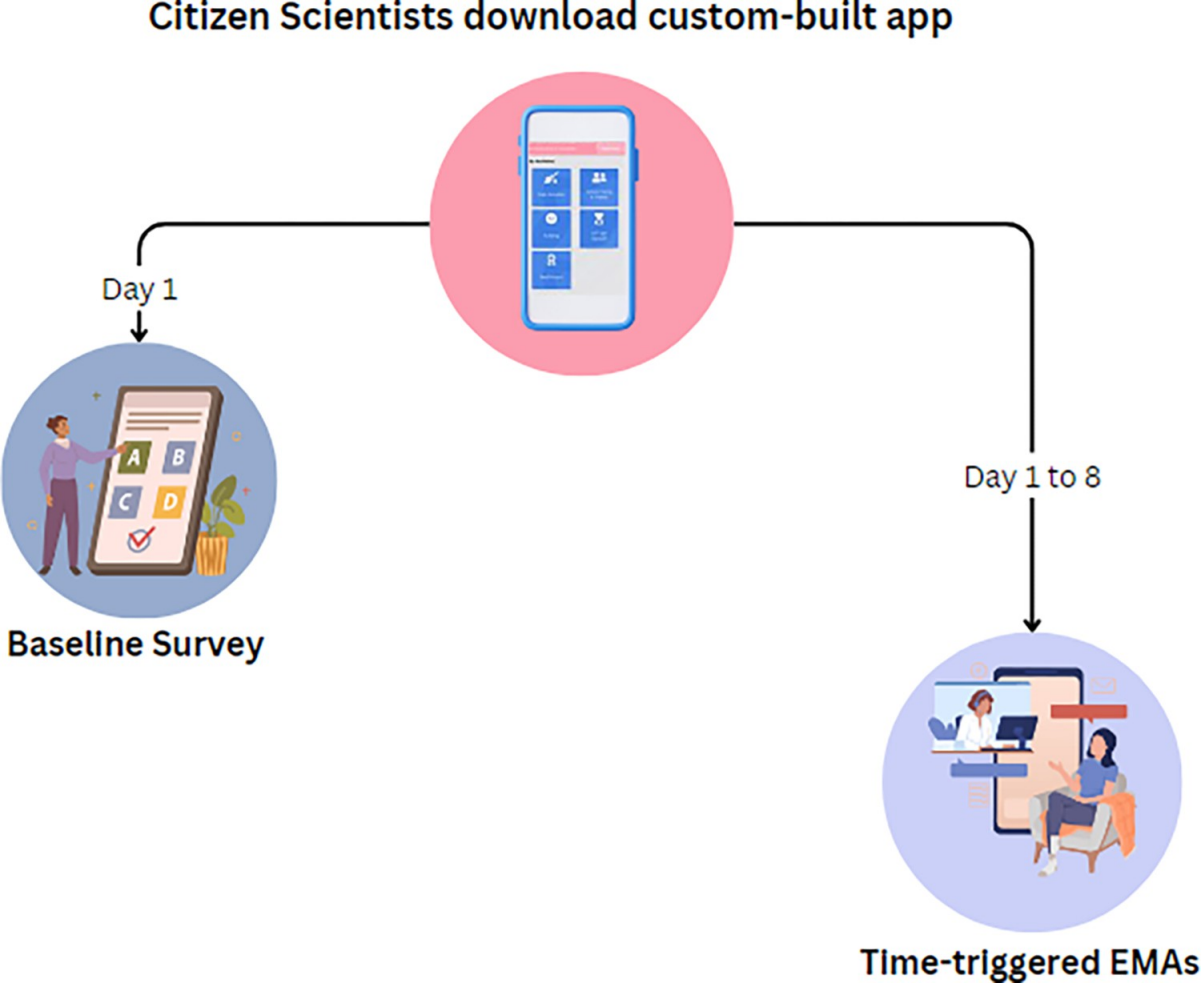
Deployment structure of prosepective and retrospective surveys.

Recognizing the importance of accurate and timely data collection, the EMAs are designed to capture prospective smartphone use on a daily basis, hence the deployment was informed by the need to capture each day’s smartphone use (i.e., towards the end of the day) to ensure accurate measurement with minimal recall bias. These deployment decisions were again informed by the feedback and engagement with both the youth themselves and the educators in their schools. To complement this data, a modified traditional retrospective recall survey was deployed using a one-time triggered notification on day one, which also collected demographic characteristics, including gender, age, and socioeconomic status **(Fig 2).** The retrospective survey investigated the habits of youth citizen scientists’ smartphone use. Later, during a period of eight days, the app triggered daily EMAs to capture prospective smartphone use including weekdays and weekends **(Fig 2).** In addition, youth citizen scientists reported on their school environment, including available facilities and resources at their school, as well as the level of family and friends’ involvement and encouragement to perform physical activities [19].

### Participants

To achieve 90% confidence level and a 5% margin of error, a sample size calculation resulted in the sample size to be at least 161. Regina Public and Catholic School Boards were contacted for recruitment of youth citizen scientists, and in-person recruitment sessions were initiated August 31^st^, 2018, until December 31^st^ of the same year. As a result, a total of 436 youth citizen scientists were recruited from five schools (aged 13–21). During the recruitment sessions, researchers demonstrated how to use the smartphone application, and youth citizen scientists were able to download and join the study at any time during the recruitment period (August 31^st^ -December 31^st^, 2018). In addition, although all citizen scientists received a one-week pass to a community fitness centre as an incentive for their participation in the Platform, an overwhelming majority (>95%) did not utilize the pass [7].

### Inclusion criteria

**Fig 3:**
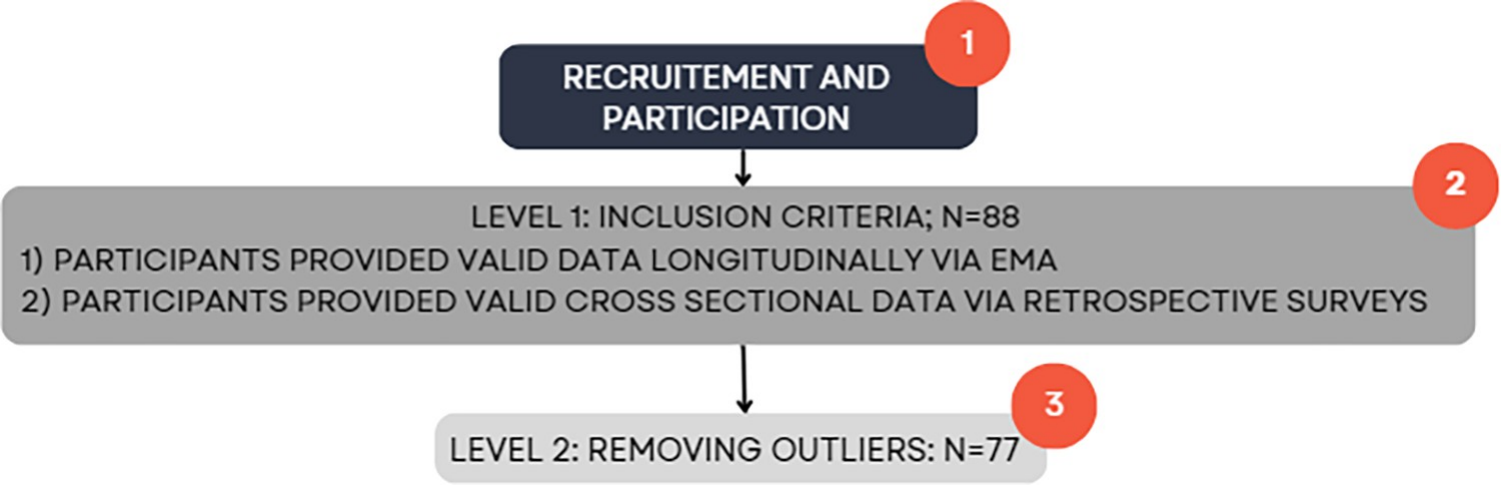
Multi-level inclusion criteria for prospective EMA and retrospective smartphone use.

All youth citizen scientists providing their data in this study were students enrolled in the participating schools, including students who were over 18 years of age as one of the schools had mature high school students (18–21). The key inclusion criteria for this study were: 1) participants provided valid data longitudinally via EMAs on at least one day 2) participants provided cross sectional data via retrospective surveys. Records with substantial missing data were excluded from the analysis to prevent bias and ensure the reliability of the results. Following the application of the inclusion criteria, 88 youth citizen scientists were included in the study. Furthermore, outliers were removed from the analysis. Subsequently, the Interquartile range (IQR) technique was implemented to identify outliers in the continuous data [20]. These outliers were removed from the dataset prior to final analyses. Consequently, this comprehensive cleaning process resulted in a refined dataset comprising 77 youth citizen scientists, enhancing the validity of the subsequent analyses and ensuring that the findings were based on robust and reliable data (**See Fig 3**).

### Ethics

The Research Ethics Boards at the Universities of Regina and Saskatchewan approved the Smart Platform’s application for research ethics approval (REB # 2017–029). Written informed consent was provided to all youth citizen scientists through the application. However, youth citizen scientists between the ages of 13–16 years were required to provide written implied consent obtained from either their caregiver(s) or parent(s) before the scheduled recruitment sessions [19].

### Measures

#### Dependent variables

Dependent variables were mean values of smartphone use on weekdays, weekends, and overall retrospective and prospective (EMAs) per day. The retrospective survey questions were deployed on day one of engagement via a custom-built application, while EMAs were deployed daily (8:00 pm-11:30 pm) and were set to expire by 01:00 am.

The 9-questions Sedentary Behaviour Questionnaire Survey questions, which is a retrospective traditional data collection tool, was modified for digital deployment on citizen scientists’ smartphones [18]. The retrospective modified survey asked questions such as: “On a typical weekday during the school year, how much time do you spend on your smartphone for Internet surfing (e.g., Facebook, Snapchat, Instagram, YouTube, Reddit, reading news, etc.)?”. From youth responses, weekday mean minutes of retrospective smartphone use was derived. Further the retrospective survey asked: “On a typical weekend during the school year, how much time do you spend on your smartphone for internet surfing (e.g., Facebook, Snapchat, Instagram, YouTube, Reddit, reading news, etc.)?”. From youth responses, weekend mean minutes of retrospective smartphone use was derived. In order to obtain overall retrospective smartphone use, the sum of mean weekday and weekend smartphone use was derived and averaged.

To ascertain prospective smartphone use, EMA questions asked: “Which of the following did you do yesterday?” With response options to include: “Watched television”, “Internet surfing using desktop/laptop/gaming device/television screen”, “Used smartphone for internet surfing”, “Texted using a phone”, “Played games on smartphones”, “Listened to music while sitting”, “Played a musical instrument while sitting”, “Did arts and crafts while sitting”, and “Drove or sat in a car/bus.”, “How many minutes did you spend doing this activity?”, and “With whom did you do this activity?” From these responses, weekend mean minutes of prospective smartphone use was derived. To obtain overall prospective smartphone use, a sum of weekday mean minutes of prospective smartphone use and weekend mean minutes of prospective smartphone use was derived and averaged. Both retrospective and prospective overall, weekday, and weekend mean minutes of smartphone use were the primary dependent variables of this study.

#### Independent variables

The independent variables encompassed Physical activity strength, school physical activity environment, and family and friends’ involvement in youth physical activity. Detailed information on all questions, categories, and dichotomized responses can be found in **S1 Appendix**.

### Data and risk management

Data were encrypted before being saved on smartphones and before being sent to secure servers when a device established a Wi-Fi connection to protect confidentiality. The app’s permissions were controlled to avoid accessing any personal data on their smartphones (e.g., contact list or network sites visited). Media Access Control (MAC), a network data transfer policy based on a basic encryption method that cannot be reversed, was used for anonymization, and to secure the information of citizen scientists. During the process of obtaining informed consent, risks and privacy management alternatives were clearly communicated to the citizen scientists [19].

### Data analysis

Characteristics of the youth citizen scientists were reported using descriptive statistics. Pearson correlation was used to assess at the association between smartphone use reported retrospectively and prospectively on weekdays and weekends. A paired sample t-test was conducted to explore if there exists a difference in mean minutes/day of retrospective smartphone use, and mean minutes/day of prospective smartphone use reported on weekday and weekend. Additionally, this study investigates if there exists a difference in mean minutes of smartphone use per day retrospectively and prospectively based on gender of youth citizen scientists. Multiple linear regression models were developed to explore the contextual and behavioural factors associated with retrospective and prospective smartphone use among youth citizen scientists. Statistical analyses were conducted using Python statistical software with a significance level of p < 0.05.

## Results

**Table 1.** presents the results of the descriptive analysis conducted on the final dataset provided by 77 youth citizen scientists. Approximately 67.10% of the participants were female, and 64.47% did not have a part-time job. Additionally, only 35.06% of the youth reported participating in a sports team at their school, while around 37.66% reported engaging in muscle training activities for four days or more (**Table 1**).

**Table 1:** Summary of characteristics of youth citizen scientists (n = 77).

Variable	
**Dependent Variables**	**Mean [minutes/day] (SD)**
Overall retrospective survey smartphone use	218.28 (140.29)
Overall prospective EMA smartphone use	53.5 (38.87)
Weekday retrospective survey smartphone use	214.01 (133.25)
Weekday prospective EMA smartphone use	54.10 (41.98)
Weekend retrospective survey smartphone use	211.50 (153.09)
Weekend prospective EMA smartphone use	68.80 (46.88)
**Independent Variables**	
**Sociodemographic variables**	**Mean (SD)**
Age in years	15.45 (1.56)
Gender	**Frequency (%)**
Male	22 (28.95)
Female	51 (67.10)
Transgender / Other / Prefer not to disclose	3 (3.95)
Total	76 (100)
Part time Job	
Yes	27 (35.53)
No	49 (64.47)
Total	76 (100)
**School Physical Activity (PA) environment**	
Participating in sports team at school	
No	50 (64.93)
Yes	27 (35.06)
Total	77 (100)
**Physical Activity Strength**	**Mean [minutes/day] (SD)**
Moderate to vigorous activity per day	94.96 (82.33)
Muscle training in past 7 days	**Frequency (%)**
Less than 4 days	48 (62.34)
4 days or more	29 (37.66)
Total	77 (100)
**Social support for physical activity**	
Family members in household encourages PA	
Discourage/neutral	20 (25)
Encourage	57 (75)
Total	77 (100)
Friends who are physically active	
Less than two	19 (24.68)
Two or more	58 (75.32)
Total	77 (100)

There was a significant difference between overall (weekdays+weekend days) mean minutes of smartphone use/day reported retrospectively (218.28 minutes/day) and prospectively (53.50minutes/day) (**Table 2**). Similarly, youth also reported more smartphone use on both weekdays and weekend days retrospectively using the traditional surveys (214.01minutes/day; 211.50minutes/day), compared to smartphone use reported prospectively via EMAs (54.10minutes/day; 68.80minutes/day). This pattern continued among both females and males **(Table 3).** There was a significant difference between overall (weekdays+weekend days), weekdays, and weekends mean smartphone use reported retrospectively (240.14 minutes, 255.90 minutes, and 266.47 minutes) and prospectively (51.84 minutes, 50.38 minustes, and 57.13 minutes) among female youth citizen scientists (p<0.001). This was also observed in overall (weekdays+weekend days) and weekdays mean smartphone use reported retrospectively (144.20 minutes, 171.32 minutes) and prospectively (64.41 minutes, 61.89 minutes) among male youth citizen scientists (p<0.001). However, among male citizen scientists, there was no significant difference between weekend mean smartphone use reported retrospectively and prospectively.

**Table 2:** Paired sample t-test result showing the differences between smartphone use retrospectively and prospectively.

Overall	Min, Max	Mean	Mean difference (95% CI)	t-value	df	N	p-value
Minutes of smartphone use reported retrospectively	7.5, 615.0	218.28	164.78 (131.74, 197.82)	9.93	76	77	<0.001
Minutes of smartphone use reported prospectively	0.0, 159.375	53.50					
**Weekday**							
Minutes of smartphone use reported retrospectively	45, 570	214.01	159.91 (126.85, 192.98)	9.64	70	71	<0.001
Minutes of smartphone use reported prospectively	1.0, 175.62	54.10					
**Weekend**							
Minutes of smartphone use reported retrospectively	0, 690	211.50	142.70 (82.91, 202.48)	4.88	29	30	<0.001
Minutes of smartphone use reported prospectively	1.0, 180.0	68.80					

**Table 3:** Paired sample t-test result showing the differences between smartphone use retrospectively and prospectively based on gender.

Overall	Female							Male						
	Min, Max	Mean	Mean difference (95% CI)	t-value	df	N	p-value	Min, Max	Mean	Mean difference (95% CI)	t-value	df	N	p-value
Overall smartphone use reported retrospectively	45.0, 525.0	240.14	188.30 (150.80, 225.81)	10.08	52	53	<0.001	7.5, 360.0	144.20	79.79 (33.13, 126.46)	3.56	21	22	<0.001
Overall smartphone use reported prospectively	0.0, 150.0	51.84						1.0, 193.54	64.41					
**Weekday**														
Weekday smartphone use reported retrospectively	45, 510	225.90	175.52 (137.01, 214.03)	9.16	49	50	<0.001	45, 480	171.32	109.43 (49.96, 168.90)	3.87	18	19	<0.001
Weekday smartphone use reported prospectively	1.0, 150.0	50.38						10.0, 175.62	61.89					
**Weekend**														
Weekend smartphone use reported retrospectively	75, 690	266.47	209.34 (127.55, 291.14)	5.43	16	17	<0.001	0, 360	138.75	42.73 (-36.74, 122.19)	1.18	11	12	0.25
Weekend smartphone use reported prospectively	1.33, 120.0	57.13						1.0, 238.33	96.02					

After controlling for age, results of the multiple linear regression models exploring social and contextual factors associated with overall, weekday, and weekend smartphone use reported via retrospectively surveys and prospective EMAs are portrayed in tables 4, 5, and 6, respectively. Within the overall retrospective model (**Table 4 –Model 1**), youth citizen scientists who reported having a part-time job (β = 139.64, 95% confidence interval [CI] = 34.759, 244.519, p-value = 0.010) reported more smartphone use compared to youth citizen scientists who did not have part-time jobs. In contrast, youth citizen scientist who had more than two friends who are physically active (β = -114.72, 95%[CI] = -208.872, -20.569, p-value = 0.018) reported less smartphone use in comparison to youth who had less than two friends that were physically active. However, none of these relationships were found to be significant in the prospective EMA model (**Table 4-Model 2**).

**Table 4:** Linear Regression models of overall smartphone use reported retrospectively vs. prospectively.

Variables	Retrospective (Model 1)		Prospective (Model 2)	
	Standardized Coefficients Beta	p-value (95% CI)	Standardized Coefficient Beta	p-value (95% CI)
(constant)	120.011	0.696 (-489.905, 729.927)	83.230	0.295 (-74.241, 240.702)
**Sociodemographic variables**				
Part-time job: Yes (Ref)				
Not having a part time job	139.639	0.010*** (34.759, 244.519)	-2.8105	0.843 (-31.114, 25.493)
Gender: Female (Ref)				
Male	-65.736	0.176 (-161.655, 30.183)	-4.4359	0.729 (-29.896, 21.025)
**Physical Activity Strength**				
Moderate to vigorous physical activity per day	0.0236	0.949 (-0.716, 0.763)	0.0673	0.372 (-0.082, 0.217)
Muscle training: less than 4 days (Ref)				
Muscle Training- 4 days or more	26.9249	0.547 (-61.796, 115.645)	-8.5956	0.483 (-32.941, 15.749)
**School PA environment**				
Participating in a sports team: No (Ref)				
Participating in a sports team	-40.2435	0.406 (-136.261, 55.774)	10.5313	0.435 (-16.212, 37.275)
**Family and Friends involvement in PA**				
Family encouragement for physical activity: Discourage/neutral (Ref)				
Family encouragement for physical activity: Encourage	-37.8963	0.472 (-142.609, 66.817)	-16.9656	0.233 (-45.127, 11.196)
Number of physically active friends: Less than two (Ref)				
Two or more physically active friends	-114.7206	0.018*** (-208.872, -20.569)	0.0470	0.997 (-25.590, 25.684)

*** *p < 0*.*05* all regression models controlled for: age

In the weekday retrospective regression model (**Table 5 –Model 3**), youth who reported not having a part-time job (β = 108.5, 95%[CI] = 10.727, 206.264, p-value = 0.03) were significantly associated with more minutes of smartphone use compared to youth who had a part-time job. This association was not found to be significant in the prospective model (**Table 5 –Model 4**).

**Table 5:** Linear regression models of weekday smartphone use reported retrospectively vs. prospectively.

Variables	Retrospective (Model 3)		Prospective (Model 4)	
	Standardized Coefficients Beta	P-value (95% CI)	Standardized Coefficient Beta	P-value (95% CI)
(constant)	137.315	0.635 (-438.387, 713.017)	59.615	0.509 (-120.031, 239.260)
**Sociodemographic variables**				
Part-time job: Yes (Ref)				
Not having a part time job	108.4959	**0.03**0*** **(**10.727, 206.264)	-0.0641	0.997 (-31.019, 30.890)
Gender: Female (Ref)				
Male	-48.4754	0.279 (-137.118, 40.167)	-5.5225	0.694 (-33.447, 22.403)
**Physical Activity Strength**				
Moderate to vigorous physical activity per day	0.2181	0.514 (-0.446, 0.882)	0.0777	0.320 (-0.077, 0.233)
Muscle training: less than 4 days (Ref)				
Muscle Training- 4 days or more	-0.3539	0.993 (-84.120, 83.412)	-10.6475	0.504 (-36.150, 14.855)
**School PA environment**				
Participating in a sports team: No (Ref)				
Participating in a sports team	-73.0199	0.112 (-163.566, 17.526)	8.4913	0.549 (-19.669, 36.651)
**Family and friends’ involvement in PA**				
Family encouragement for physical activity: Discourage/neutral (Ref)				
Family encouragement for physical activity: encourage	3.7939	0.940 (-96.426, 104.014)	-9.9383	0.504 (-39.475, 19.598)
Number of physically active friends: Less than two (Ref)				
Two or more physically active friends	-45.1814	0.320 (-135.178, 44.815)	3.3507	0.807 (-23.897, 30.598)

*** *p < 0*.*05* all regression models controlled for: age

In the weekend retrospective model (**Table 6 - Model 5**), youth citizen scientists who did not have a part-time job (β = 127.28, 95%[CI] = 20.831, 233.730, p-value = 0.020) reported significantly greater smartphone use compared to youth who did not have a part-time job. Male youth citizen scientist reported significantly lower smartphone use (β = -121.44, 95%[CI] = -220.227, -22.654, p-value = 0.017). Similarly, youth who reported having more than two friends were found to be associated with less smartphone use (β = -140.850, 95%[CI] = -236.326, -45.374, p-value = 0.004) as compared to youth who did not report participating in school sports team. However, these significant associations were not found to be significant in the prospective EMA model (**Table 6 –Model 6**).

**Table 6:** Linear regression models of weekends smartphone use reported retrospectively vs. prospectively.

Variables	Retrospective (Model 5)		Prospective (Model 6)	
	Standardized Coefficients Beta	p-value (95% CI)	Standardized Coefficient Beta	p-value (95% CI)
(constant)	293.608	0.346 (-323.696, 910.912)	286.039	0.132 (-94.853, 666.931)
**Sociodemographic variables**				
Part-time job: Yes (Ref)				
Not having a part time job	127.281	0.020*** (20.831, 233.730)	30.102	0.388 (-41.522, 101.727)
Gender: Female (Ref)				
Male	-121.440	0.017*** (-220.227, -22.654)	-25.891	0.478 (-101.143, 49.362)
**Physical Activity Strength**				
Moderate to vigorous physical activity per day	-0.021	0.956 (-0.767, 0.726)	-0.044	0.839 (-0.500, 0.411)
Muscle training: less than 4 days (Ref)				
Muscle Training- 4 days or more	40.213	0.376 (-49.805, 130.231)	-49.1449	0.237 (-133.779, 35.490)
**School PA environment**				
Participating in a sports team: No (Ref)				
Participating in a sports team	-25.333	0.607 (-123.125, 72.460)	38.246	0.214 (-24.307, 100.799)
**Family and Friends involvement in PA**				
Family encouragement for physical activity: Discourage/neutral (Ref)				
Family encouragement for physical activity: encourage	-74.232	0.167 (-180.430, 31.966)	-22.146	0.510 (-91.514, 47.222)
Number of physically active friends: Less than two friends (Ref)				
Two or more physically active friends	-140.850	0.004*** (-236.326, -45.374)	-3.810	0.895 (-63.994, 56.375)

*** *p < 0*.*05* all regression models controlled for: age

## Discussion

The objective of this study was to compare smartphone use reported via retrospective surveys and prospective EMAs by engaging with youth citizen scientists via their own smartphones. The study also investigated the behavioural, and contextual factors that are associated with smartphone use reported via retrospective surveys and prospective EMAs. The primary findings of this study demonstrate that there is a significant difference in smartphone use reported retrospectively (via validated surveys) and prospectively (via EMAs). While evidence generally indicates that retrospective data collection is prone to bias irrespective of overestimation or under-estimation [21–23], to our knowledge, no research has been carried out to compare retrospective recall surveys and prospective EMAs to assess smartphone use within the same cohort of participants.

While there is some evidence of over-estimation of sedentary behaviours when reporting retrospectively [21], and discrepancies in reporting smartphone use between males and females [24], our study is the first digital epidemiological investigation that compared smartphone use reporting retrospectively and prospectively among the same cohort of youth. There is some evidence that individuals who are more socially engaged might over-estimate their smartphone use, while individuals who under-estimate their smartphone use may have lost track of time [22]. In general, evidence indicates that prospective measurement of behaviours using EMAs is less susceptible to recall bias [18,25].

Although the World Health Organization guidelines indicate that youth aged 15–18 should not spend more than two hours of screentime per day [26], current evidence indicates that youth across the world spend significantly more time than two hours/day on screens [27,28]. The uncontrolled screentime has detrimental effects on both physical and mental health leading to increased risk of non-communicable chronic diseases such as obesity, diabetes, heart diseases, reduced sleep quality as well as stress, anxiety, and depression [29]. Furthermore, the screentime guidelines do not specify the time that youth should spend on mobile devices, particularly smartphones, which most youth have access to in this digital age [30,31]. This gap in guidelines needs to be addressed, particularly because smartphones are the primary devices of day-to-day functioning, and excessive smartphone use is associated with poor youth health outcomes [5,15,32]

However, to understand associations between smartphone use and youth health outcomes, it is imperative to obtain valid and reliable data from the youth, which, again ironically is possible via smartphones themselves due to their near universal usage among youth [5,33]. This study utilized the Smart platform [19], a digital citizen science platform that engaged youth as citizen scientists to obtain both retrospective and prospective smartphone use via their own smartphones [7].

This approach also enabled our team to obtain relevant data on behavioral, contextual, and demographic factors to investigate the association of these variables with both retrospective and prospective smartphone use among youth. Moreover, as evidence indicates that smartphone use and screentime in general varies between weekdays and weekend days [5,32] this study explored the association of behavioural and contextual factors with overall (weekday+weekend day) youth smartphone use, as well as weekday and weekend day smartphone use reported by both retrospective surveys and prospective EMAs.

One consistent finding was that there were no significant associations between socio-demographic and contextual factors with overall, weekday, and weekend day smartphone use reported via EMAs.

However, retrospective models showed some common associations between sociodemographic and contextual factors with overall, weekday and weekend day smartphone use. Youth who reported that they did not have a part-time job also reported significantly more overall, weekday, and weekend day smartphone use in comparison with youth who had a part-time job—a finding that is consistent with some existing evidence [34], and potentially an indication that youth, due to the nature of part-time work they do, do not have access to smartphones consistently.

Likewise, a systemized review reported that having friends who are physically active is associated with increasing the level of physical activity of their peers and lowering sedentary behaviour [35]. This finding is consistent with the findings of our study, which also showed that youth who have more than two friends who are physically active reported lower smartphone use on weekends.

Irrespective of the direction of association of contextual and sociodemographic variables, there are some important aspects that must be highlighted in understanding smartphone use in general, and particularly among youth. First, all significant findings were depicted in the retrospective smartphone use reporting models, and no significant findings were depicted in the prospective smartphone use reporting (EMAs). This in itself is a major finding that emphasizes the rationale for conducting this study–it is critical that more accurate measures of reporting be standardized to understand smartphone use because retrospective self-reporting data collection tools are prone to bias and misclassification [15,36,37].

Second, it is imperative to appreciate the complexity of smartphone use, which includes various behaviours and motivations, such as social media use, gaming, and texting, among others, which have varying associations with health outcomes [38–41]. To capture these effectively and accurately, it is critical to move towards prospective measures such as EMAs which minimize recall bias, particularly when the reporting is complicated by the wide range of behaviours [42]. Objective measures provide accurate overall smartphone use estimates [7], however, capturing the variation in smartphone use ethically, while simultaneously preserving privacy can be challenging [43]. This challenge may arise from the potential extraction of personal and sensitive information from mobile device sensor data [44]. Extracting personal and sensitive information from mobile device sensor data may potentially also breach user privacy [45]. These challenges must be navigated carefully to ensure ethical standards are maintained. Moreover, objective measures can indeed provide a more accurate and detailed breakdown of smartphone use, including specific app usage. However, objective data does need to be triangulated with subjective information from citizens, which will enable validation of data [46]. The objective measures are ideal for capturing volume, type, and frequency of smartphone use if the privacy concerns can be mitigated [47].

Finally, the contextual and sociodemographic associations of smartphone use also have to be captured prospectively to minimize bias, while again, appreciating the complexity of smartphone access itself [12]. For instance, male youth citizen scientists reported less smartphone use compared to female youth citizen scientists, which is consistent with the findings of other studies [24].

Perhaps more importantly, the approaches we use to capture digital data ethically from citizens must be revisited. In scientific research settings, the power often lies with those who have knowledge such as researchers, which has been an obstacle for meaningful data collection, particularly if the data are sensitive and need to be obtained via citizen-owned devices [47]. Digital citizen science is an approach that can democratize science through engagement with citizens ethically and directly to obtain prospective big data [48–50]–an approach that was utilized while collecting data from youth in this study via their own smartphones [7,51–54].

Appropriate measurement of smartphone use among youth is critical to not only inform potential smartphone use recommendations [15], but to also determine the appropriate use for digital health interventions [16]. These interventions have the potential to address mental health illnesses at a reduced cost and improve access to resources [33].

## Strengths and limitations

No studies have compared smartphone use among youth using validated retrospective traditional surveys and prospective EMAs. The strengths of this study include utilizing citizen science approaches, where youth used their own devices to engage with the researchers. The key limitation is that the smartphone use reported in this study does not delineate between the different types of smartphone use informed by varied motivations–social media, texting, gaming, among others. Another limitation is that data were collected during one season even though there is evidence that screentime and sedentary behaviours are influenced by changes in weather [55,56]. Additionally, failing to meet the required sample size is a significant limitation of this study. Nevertheless, the unique nature of this study sets a foundation for future investigations, encouraging further exploration and validation. Future studies should not only measure different types of smartphone use, but also aim to capture objective data to improve accuracy of reporting, while taking seasonality into consideration [57,58].

## Conclusion

The findings of this study have implications for appropriately understanding and monitoring smartphone use in the digital age among youth. EMAs can potentially minimize recall bias of smartphone use among youth, and other behaviours. More importantly, digital citizen science approaches that engage large populations of youth using their own smartphones can transform how we ethically monitor and mitigate the impact of excessive smartphone use.

## Supporting information

S1 Appendix Description of Independent variables used in the study. (DOCX)
