# Supplementary material for: How can digital citizen science approaches improve ethical smartphone use surveillance among youth: Traditional surveys versus ecological momentary assessments
Source: PLOS Digit Health. 2024 Nov 11;3(11):e0000448. doi: 10.1371/journal.pdig.0000448 (PMC11554190; doi:10.1371/journal.pdig.0000448)
Supplement: S1 Appendix — (DOCX) [file pdig.0000448.s001.docx]

**S1 Appendix. Description of Independent variables used in the study**

| **Independent variables** | **Categories** | **Dichotomized codes** |
| --- | --- | --- |
| **Sociodemographic** |  |  |
| Age | Continuous variable | N/A |
| “What is your gender?” | “Male”, “Female”, “Transgender”, “Other (please specify)”, or “Prefer not to disclose” | “Female”, “Male”, or “Transgender/other (please specify)/prefer not to disclose” |
| “Do you have a part-time job?” | “yes” and “no” | “yes” and “no” |
| **Physical activity** |  |  |
| “How many minutes did you spend performing moderate to vigorous physical activity on each day of the week?”. | Continuous variable | N/A |
| “On how many days in the last 7 days did you do exercise to strengthen or tone your muscles (e.g., push-ups, sit-ups, or weight-training)?” | “0 days,” “1”, “2”, “3”, “4”, “5”, “6”, or “7 days”. | “less than four days”, and “four days or more”. |
| **School PA environment** |  |  |
| “Do you participate in competitive school sports teams that compete against other schools (e.g., junior varsity or varsity sports)?” | “Yes”, “No” or “None offered” | Yes: yes  No: No, none offered |
| **Family and friends’ involvement in PA** |  |  |
| “How much do your parents, stepparents, or guardians encourage you to be physically active?” | “Strongly encourage”, “Encourage”, “Do not encourage nor discourage”, “Discourage”, or “Strongly discourage”. | “Discourage/neutral”: “Do not encourage nor discourage”, “Discourage”, or “Strongly discourage”.  “Encourage”: “Strongly encourage”, “Encourage” |
| “How many of your closest friends are physically active?” | “None of my friends”, “1”, “2”, “3”, “4”, or “5 of my friends.” | Less than two of my friends”: “None of my friends”, and “1 of my friends”  “Two or more of my friends”: “2”, “3” ,“4”, or “5 of my friends.” |
